# APOE and FABP2 Polymorphisms and History of Myocardial Infarction, Stroke, Diabetes, and Gallbladder Disease

**DOI:** 10.1155/2011/896360

**Published:** 2011-09-18

**Authors:** Ikuko Kato, Susan Land, Jill Barnholtz-Sloan, Richard K. Severson

**Affiliations:** ^1^Karmanos Cancer Institute, School of Medicine, Wayne State University, 4100 John R. Street, Detroit, MI 48201, USA; ^2^Department of Pathology, School of Medicine, Wayne State University, Detroit, MI 48201, USA; ^3^Department of Obstetrics and Gynecology, School of Medicine, Wayne State University, Detroit, MI 48201, USA; ^4^Case Comprehensive Cancer Center, Case Western Reserve University School of Medicine, Cleveland, OH 44106, USA; ^5^Department of Family Medicine and Public Health Sciences, School of Medicine, Wayne State University, Detroit, MI 48201, USA

## Abstract

Dysfunctional lipid metabolism plays a central role in pathogenesis of major chronic diseases, and genetic factors are important determinants of individual lipid profiles. We analyzed the associations of two well-established functional polymorphisms (*FABP2* A54T and *APOE* isoforms) with past and family histories of 1492 population samples. *FABP2-T54* allele was associated with an increased risk of past history of myocardial infarction (odds ratio (OR) = 1.51). Likewise, the subjects with *APOE4*, compared with *E2* and *E3*, had a significantly increased risk of past history myocardial infarction (OR = 1.89). The OR associated with *APOE4* was specifically increased in women for past history of myocardial infarction but decreased for gallstone disease. Interactions between gender and *APOE* isoforms were also significant or marginally significant for these two conditions. *FABP2-T54* allele may be a potential genetic marker for myocardial infarction, and *APOE4* may exert sex-dependent effects on myocardial infarction and gallbladder disease.

## 1. Introduction

Dysfunctional lipid metabolism plays a central role in pathogenesis of major chronic diseases, including cardiovascular diseases, insulin-independent diabetes, and gallbladder disease [1, 2]. While environmental factors, such as diet, are important determinants of circulating lipid concentrations, heritabilities of lipid profiles have been well elucidated in twin studies [3–6]. Because of the well-established association between circulating cholesterol levels and cardiovascular diseases, genes involved in cholesterol synthesis and transport have been more extensively studied than the others involved in long-chain fatty acids (LCFAs) and phospholipids [2, 7–11]. 

Among a number of genetic loci that have been examined thus far, APOE isoforms are the first common genetic polymorphisms that were linked to cardiovascular diseases (CVDs) and Alzheimer's disease [9, 12–14]. ApoE is a multifunctional protein that is synthesized by the liver and several peripheral tissues and cell types, including macrophages, and a major component of several classes of plasma lipoproteins including triglyceride-rich very-low-density lipoprotein (VLDL) [13]. The protein is involved in the efficient hepatic uptake of lipoprotein particles, stimulation of cholesterol efflux from macrophage foam cells in the atherosclerotic lesion, and the regulation of immune and inflammatory responses and thus has key roles in lipid transport in the plasma and in the central nervous system as well as in responses to the dietary fat content and fatty acid compositions [13–15]. Three isoforms, *E2*,* E3*, and *E4*, are defined by combinations of amino acid (cysteine/arginine) substitutions at codons 112 and 158 [12–14]. The most recent meta-analysis by Bennet et al. demonstrated approximately linear relationships of *APOE* genotype (*E2* through *E4*) with both LDL cholesterol levels and coronary risk, highlighting the effect of *E2* [16], while two earlier analyses emphasized the adverse effect of *E4* on coronary risk [17, 18]. Some studies also suggest that genetic effects of *APOE* on several lipid parameters may be modified by gender [19–25]. *FABP2* encodes intestinal fatty acid binding protein, and its alanine (A)/threonine (T) substitution at codon 54 in exon-2 [26] has received research interest as *T54*-containing FABP2 protein has greater ability to transport LCFA than A54-containing protein [27]. T54 allele has been associated with higher plasma triglycerides [28–30] and free fatty acids levels [31], a higher postprandial lipemic response [32, 33], and higher postprandial glucose and insulin levels [26, 34, 35], as well as with CVD and type 2 diabetes in some studies [36–41]. Due to their extensively studied roles in responses to dietary fat intake as well as to the established functional effect in vitro [8, 9, 14, 15, 27–35], these two polymorphisms were originally chosen for the parent study to elucidate the association with colorectal cancer [42], although the list of genes is now growing, including *APOA5* and *LPL* [2, 9–11], owing to advance in genotyping technologies. 

These polymorphisms are likely to have implications in multiple chronic conditions related to lipid metabolism, but most of the published studies to date have focused on single conditions. Using the data from a population-based case-control study [42], we had an opportunity to address if these functional polymorphisms are associated with histories of myocardial infarction, stroke, diabetes, and gallbladder disease.

## 2. Methods

### 2.1. Study Design

This study was secondary data analysis of the control subjects who participated in a population-based case-control study for colorectal cancer in Metropolitan Detroit, Mich, USA. Details concerning the ascertainment, recruitment, and characteristics of the study subjects of the parent study have been described elsewhere [42]. The study was approved by Wayne State University Human Investigation Committee and all subjects gave written informed consent to participate in the study. In brief, eligible subjects were between 45 and 80 years of age at time of ascertainment, with a working telephone and no prior history of any invasive cancer, in situ colorectal cancer, or colectomy and were recruited through random digit dialing. These subjects consisted of 57% of females, 43% of males, 26.5% of African Americans, 69.2% of Non-Hispanic Caucasians, and 4.3% of other racial groups. The subjects were interviewed over the telephone using structured questionnaires regarding their usual diet and other risk factors for colorectal cancer (e.g., demographics, smoking, physical activity, medication/supplement use, and own and family history of major chronic diseases) for the time-period preceding cancer diagnosis for the cases (approximately 2 years prior to the interview). Specifically, a semiquantitative food frequency questionnaire (FFQ), Block 98.2 (Block Dietary Data Systems, Berkeley, Calif), that was validated against multiple diet records [43, 44] was used to estimate daily nutrient intake. This questionnaire was chosen based on its superiority to categorize individuals on energy from fat as compared to the Willett instrument [45] as dietary fat was a main focus of the study. The residual method described by Willett and Stampfer was used to calculate energy-adjusted nutrient intake [46]. The study participants provided either peripheral blood (71%) or buccal cell samples (29%) for TaqMan genotyping for *ApoE* and *FABP2* polymorphisms. Both polymorphisms were found to be in Hardy-Weinberg equilibrium using *P* = 0.05 as the threshold.

### 2.2. Statistical Analysis

Out of the 1547 controls originally consented to the study, 1492 with all study parameters were included in this analysis. The following histories of diseases were queried for subjects themselves as well as for their first degree of family members (parents, children, and siblings): myocardial infarction/heart attack, stroke and transitional ischemic attack, diabetes, and gallstone/gallbladder surgery. Odds ratios (ORs) and 95% confidence intervals (CIs) for history (own, family, or both) of these diseases associated with *FABP2* and *APOE* polymorphisms were estimated using an unconditional logistic regression model [47], adjusting for selected covariates, which included demographic variables (age, sex, race (African American versus others), and educational level), family size (numbers of own siblings and children), common risk factors for major chronic diseases (pack-years of cigarette smoking, body mass index (body weight (kg)/body height (m)^2^), and alcohol intake), and dietary covariates (energy-adjusted saturated fat, cholesterol, dietary fiber, and total calcium intakes) that were known risk factors for the diseases of interest and differed by either *FABP2* or *APOE* genotype. For the *FABP2* polymorphism, the *AA* genotype was used as the reference to calculate the ORs for *AT*,* TT*, or those combined. *E3* homozygotes were used as the reference for *APOE* to calculate ORs associated with *E2 *(*E2/E2*,* E2/E3, *and *E2/E4*) and *E4* (*E4/E4* and *E4/E3*) isoforms, while the ORs were also calculated for *E4* compared with *E2* and *E3* combined. The decision to include *E2/E4 to E2 *was based on the meta-analysis by Bennet et al. that clearly demonstrated that total and LDL cholesterol levels were lower in the *E2/E4* genotype than in the wild-type *E3/E3* group [16]. Tests for linear trend in the logit of risk associated with these ordinal categorical genotypes were performed using equally spaced scores to the categories. In addition, the ORs were calculated stratified by gender, and the interactions between the polymorphisms and gender were tested by including their multiplicative interaction terms. All statistical analyses were performed using SAS version 9.

## 3. Results

Overall, a total of 452 subjects reported previous diagnosis or treatment for one of the diseases specified above, while 1095 reported family history of these diseases. Out of these 452, 357 had additional family history. Subjects who had been diagnosed with these diseases were older than those who had not. While diabetes and gallbladder disease were common as past histories, heart attack was most prevalent as a family history (Table 1). 

As shown in Table 2, the subjects who carried at least one *FABP2*-*T54* allele were at an increased risk of past history of myocardial infarction (OR = 1.51, 95% CI: 1.01–2.27). There was no difference between homozygotes and heterozygotes. The risk of myocardial infarction was further increased if they had family history (OR = 1.93, 95% CI: 1.15–3.23). For other diseases, there were no consistent associations with the T54 allele, except an increased risk of family history of diabetes (*P *value for trend = 0.029). Likewise, the subjects with the *APOE4 *genotypes had a significantly increased risk of past history of myocardial infarction compared with *E2 *and *E3* combined (OR = 1.89; 95% CI: 1.24–2.88) (Table 3). The similar trend was observed for the subjects with both past and family histories of myocardial infarction, but it remained marginally statistically significant (OR = 1.64, 95% CI: 0.97–2.80). There were no statistically significant associations between *APOE* genotypes and the rest of the diseases. Although the information was available only for the subjects themselves, the prevalence of high blood cholesterol or use of cholesterol-reducing medication increased progressively from *E2* through *E4* with a significant linear trend (*P* = 0.002) (data not shown). 

Table 4 presents gender-specific ORs for past history of these conditions according to the *APOE* polymorphism. The increasing risk of myocardial infarction from *E2* through *E4* was only observed in women. Compared with *E3* homozygotes, the ORs associated with *E2* and *E4* were 0.52 (95% CI: 0.17–1.62) and 1.93 (95% CI: 1.00–3.70), respectively, in women, while in men both ORs were equally elevated. As a result, the interaction between gender and *APOE* isoforms was statistically significant (*P* = 0.01). There were no statistically significant differences in the association of *APOE* with diabetes by gender. The association of *APOE *isoforms with gallbladder disease was the opposite in men and women. In women the OR was the lowest with *E4* (0.67, 95% CI 0.43–1.06) and the highest with *E2* (1.28, 95% CI: 0.79–2.08) (*P* value for trend = 0.025), but in men it was the highest with *E4 *(1.14, 95% CI: 0.58–2.26) and the lowest with *E2 *(0.58, 95% CI: 0.22–1.58) (*P* value for trend = 0.244). The interaction with gender was marginally statistically significant (*P* = 0.088). There were no significant interactions between *FABP2* polymorphism and gender for any medical conditions (data not shown).

## 4. Discussion

The results of the present study are consistent with those in a few other studies that reported the significant effect of *FABP2-T54* on the risk of myocardial infarction or coronary heart disease with the ORs ranging from 1.41 to 2.50 [36–38]. It is interesting to note that the positive associations in these earlier studies were found in patients who were diagnosed with metabolic syndrome or had one of the conditions of the syndrome. Although we did not collect the information about hypertension, 59.5% of our study subjects had at least one condition that satisfied or was indicative of the criteria for metabolic syndrome, that is, obesity (BMI (kg/m^2^) ≥ 30), history of diabetes, or a history of high blood cholesterol (a surrogate marker for atherogenic dyslipidemia) [48]. Thus, these results suggest that other cardiovascular risk factors may be important effect modifiers of the *FABP2* polymorphism and warrant further studies in populations with diverse risk profiles for CVD.

We also confirmed the association between *APOE *polymorphism and risk of myocardial infarction reported by many others [16–18], especially for *E4* genotypes. Although the latest meta-analysis showed stepwise increases in total and LDL cholesterol levels and in risk of coronary heart disease from *E2 *homozygotes, *E2* heterozygotes, *E3/E3 *wild-type, *E4/E3, *and *E4* homozygotes [16], there were not enough subjects in each of the combinations, *E2/E2 *(*N* = 11), *E2/E4 *(*N* = 43), and *E4/E4 *(*N* = 42), to analyze them separately in our study. The exclusion of *E2/E4* from the *E2* group, however, had only nominal effects on the OR estimates associated with *E2* genotypes. For example the OR for past history of MI changed from 1.14 (0.62–2.08) to 1.11 (0.58–2.13). Furthermore, our study and others suggest that the effects of *APOE* genotypes were greater in women than in men [19–25]. APOE is primarily produced in the liver, while it has been well documented that the liver is a sexually dimorphic organ. Hepatic gene expression is often sex specific, mediated through sex-dependent activation of several liver-enriched transcription factors in response to sex-specific secretion patterns of pituitary growth hormone [49, 50]. In fact, higher circulating levels of APOE in females, female-predominant hepatic expression of fatty acid translocase and differential fatty acid compositions between males and females have been demonstrated in both humans and rodents [51–53]. Thus, effects of genetic polymorphisms may be more articulated in females through such transcriptional upregulation. This also explains the absence of the interaction between gender and *FABP2* (intestinal type), whereas sex-dimorphic effects of *FABP1* (liver-type) knockout have been noted in rodent models [54, 55]. 

Surprisingly few studies have addressed the association between the *APOE* polymorphism and risk of diabetes. However, our marginally significant inverse association with *E4* genotypes and past history of diabetes was consistent with a mouse model demonstrating that APOE deficiency abrogates insulin resistance [56] and with the observations in humans that the *E4* genotypes were associated with a decreased risk and* E2* genotypes with an increased risk of diabetes [57, 58]. On the other hand, the associations between the *FAPB2* polymorphism and diabetes remain inconsistent [59] despite the fact that the polymorphism has been associated with postprandial glucose and insulin levels [26, 34, 35]. Our study found the only modest association between *T54* allele and family history, which was not confirmed with own history and thus likely to be a chance finding. 

Excessive biliary cholesterol in conjunction with decreased bile acid output is the requisite for the formation of gallstones, particularly for cholesterol stones which account for the vast majority of this disease in Western countries and which are much more common in females than in males [60]. *APOE* plays a role not only in cholesterol metabolism but also in bile acid synthesis. Fecal bile acid output has been reported to be lowest in the subjects with *E4* allele, highest in the subjects with *E2* allele, and intermediate in the subjects with *E3 *homozygotes [61–65]. While the results of earlier studies are very mixed, some with a positive association with* E4* [66–70], others with no association [71–74], and one with an inverse association [75, 76], the current study indicates that the effects of *APOE* may also be sex dependent, that is, a significantly reduced risk in women with *E4*, but not in men. This corresponds to the *APOE* knockout mouse model where bile acid synthesis markedly varies by gender [77].

There are several limitations in this study. We realize that a certain degree of misclassification exists in self-reported past and family history of diseases, which is likely to bias the results toward a null association. As far as the medical conditions selected for this study are concerned, moderate to high agreement (0.55–0.91 as kappa statistics) has been observed between self-reports and in medical records [78–80]. In addition, more than 50% of our study subjects had some college education [42], which further ensures the quality of self-reported data. Therefore, the magnitude of underestimation of the ORs is likely to be relatively small. Another concern is the coverage of disease spectra. Fatal cases of their own could not be included in this study, whereas fatal cases in their families may have been recalled more accurately. Thus, the interpretation of the ORs for past and family histories may differ particularly for CVD. Exclusion of fatal cases as well as cases who had severe complications especially in their speech may account for the small number of past histories of stroke reported in this study. Third, these chronic conditions stay asymptomatic for a long period of time. Thus, diagnoses of such asymptomatic cases depend on their preventive care or the presence of other conditions that require routine follow-up visits to health care professionals. Accordingly, some undiagnosed cases of diabetes and gallstones and subjects who underwent cardiac bypass or angioplasty without a heart attack were classified as a negative history in this study, which would further reduce potential differences between cases and noncases. Fourth, there is significant likelihood that some of the associations observed in this study were chance findings due to multiple comparisons in terms of outcome variables, although we focused on the two well-established functional polymorphisms. Finally, we acknowledge reverse temporal associations between the disease outcomes and some of the covariates included in the model, that is, smoking, BMI, and alcohol and dietary intake, which may have changed due to the diagnosis. However, these yielded predicted directions with histories of all diseases combined, that is, significant positive associations with cigarette smoking and BMI and inverse association with alcohol and calcium intake (data not shown). Although these adjustments may not have fully controlled confounding, it is less likely that the genotype distributions were affected by these covariates.

## 5. Conclusions

Despite the limitations discussed above, the results of the present study suggest that *FBBP2-T54* allele is potential genetic marker for myocardial infarction and that the *APOE4* isoform may exert opposite effects on myocardial infarction and gallstone disease in women, increasing risk for the former and decreasing risk for the later.

## Figures and Tables

**Table 1:** The numbers of study subjects who reported past or family history of selected chronic diseases and their mean ages at study enrolment.

Diseases	Medical history					
	Own		Family		Both	
	No.	Mean age	No.	Mean age	No.	Mean age
Myocardial infarction	110	66.7	663	62.8	66	66.3
Stroke	74	68.2	444	62.6	21	66.0
Diabetes	204	64.7	573	62.1	123	64.6
Gallbladder disease	203	64.7	280	60.8	53	63.7
Any of the above	452	65.0	1095	62.4	357	65.0
None of the above	1040	61.3	397	62.5	1135	61.6

**Table 2:** Odds ratios (ORs) and 95% confidence intervals (CIs) for history of selected chronic diseases according to FABP2 genotypes.

Diseases	Genotype	Medical history								
		Own			Family			Both		
		Yes/No	OR	95% CI	Yes/No	OR	95% CI	Yes/No	OR	95% CI
Myocardial infarction	AA	51/777	1.00	—	358/470	1.00	—	27/801	1.00	—
	AT	51/509	1.55	1.02–2.35	262/298	1.14	0.92–1.42	35/525	2.04	1.20–3.46
	TT	8/96	1.33	0.60–2.98	43/61	0.94	0.62–1.43	4/100	1.30	0.44–3.87
	AT + TT	59/605	1.51	1.01–2.27	305/359	1.11	0.90–1.36	39/625	1.93	1.15–3.23
		*P* = 0.089			*P* = 0.595			*P* = 0.051		

Stroke	AA	41/787	1.00	—	258/570	1.00	—	13/815	1.00	—
	AT	30/530	1.11	0.67–1.84	158/402	0.86	0.68–1.09	8/552	—	—
	TT	3/101	0.65	0.19–2.20	28/76	0.85	0.54–1.36	0/104	—	—
	AT + TT	33/631	1.05	0.64–1.70	186/478	0.86	0.68–1.08	8/656	0.80	0.32–1.97
		*P* = 0.869			*P* = 0.220			*P* = 0.406		

Diabetes	AA	118/710	1.00	—	298/530	1.00	—	75/753	1.00	—
	AT	76/484	0.94	0.68–1.31	234/326	1.32	1.06–1.65	44/516	0.88	0.59–1.32
	TT	10/94	0.66	0.32–1.35	41/63	1.26	0.82–1.93	4/100	0.46	0.16–1.30
	AT + TT	86/578	0.90	0.66–1.24	275/389	1.31	1.06–1.63	48/616	0.82	0.55–1.21
		*P* = 0.335			*P* = 0.029			*P* = 0.168		

Gallbladder disease	AA	112/716	1.00	—	148/680	1.00	—	30/798	1.00	—
	AT	81/479	1.05	0.76–1.44	118/442	1.20	0.91–1.59	21/539	0.93	0.52–1.68
	TT	10/94	0.74	0.37–1.49	14/90	0.73	0.40–1.33	2/102	0.56	0.13–2.43
	AT + TT	91/573	1.00	0.73–1.36	132/532	1.13	0.86–1.47	23/641	0.88	0.50–1.56
		*P* = 0.710			*P* = 0.876			*P* = 0.512		

ORs were adjusted for age, sex, race, educational levels, cigarette smoking (pack-years), alcohol intake, body mass index, energy-adjusted dietary saturated fat, cholesterol, fiber and total calcium intakes, and family size. *P *values for a linear trend for the number of T-alleles.

**Table 3:** Odds ratios (ORs) and 95% confidence intervals (CIs) for history of selected chronic diseases according to APOE genotypes.

Diseases	Genotype	Medical history								
		Own			Family			Both		
		Yes/No	OR	95% CI	Yes/No	OR	95% CI	Yes/No	OR	95% CI
Myocardial infarction	*E2*	16/223	1.14	0.62–2.08	113/126	1.16	0.86–1.55	9/230	0.99	0.46–2.13
	*E3*	51/810	1.00	—	378/483	1.00	—	33/828	1.00	—
	*E4*	43/349	1.95	1.25–3.04	172/220	1.01	0.79–1.29	24/368	1.64	0.94–2.86
	*E4* versus *E2E3 *		1.89	1.24–2.88		0.98	0.77–1.24		1.64	0.97–2.80
		*P* = 0.022			*P* = 0.477			*P* = 0.119		

Stroke	*E2*	15/224	1.28	0.67–2.45	77/162	1.11	0.81–1.52	3/236	0.75	0.20–2.74
	*E3*	37/824	1.00	—	255/606	1.00	—	12/849	1.00	—
	*E4*	22/370	1.10	0.63–1.93	112/280	0.94	0.72–1.23	6/386	0.87	0.31–2.42
	*E4* versus *E2E3 *		1.03	0.61–1.76		0.92	0.71–1.19		0.93	0.35–2.49
		*P* = 0.764			*P* = 0.382			*P* = 0.904		

Diabetes	*E2*	36/203	0.98	0.64–1.51	98/141	1.09	0.81–1.47	26/213	1.35	0.81–2.23
	*E3*	121/740	1.00	—	323/538	1.00	—	64/797	1.00	—
	*E4*	47/345	0.73	0.50–1.07	152/240	1.00	0.78–1.28	33/359	1.02	0.64–1.61
	*E4* versus *E2E3 *		0.73	0.51–1.06		0.98	0.77–1.25		0.94	0.61–1.46
		*P* = 0.173			*P* = 0.645			*P* = 0.395		

Gallbladder disease	*E2*	36/203	1.09	0.72–1.67	49/190	1.20	0.83–1.74	11/228	1.38	0.66–2.90
	*E3*	121/740	1.00	—	157/704	1.00	—	29/832	1.00	—
	*E4*	46/346	0.80	0.55–1.17	74/318	1.12	0.81–1.53	13/379	0.98	0.50–1.95
	*E4* versus *E2E3 *		0.79	0.55–1.13		1.07	0.79–1.45		0.91	0.47–1.75
		*P* = 0.187			*P* = 0.866			*P* = 0.473		

ORs were adjusted for age, sex, race, educational levels, cigarette smoking (pack-years), alcohol intake, body mass index, energy-adjusted dietary saturated fat, cholesterol, fiber and total calcium intakes, and family size, *E2* includes *E2/E2*, *E2/E3*, and *E2/E4*, and *E4* includes *E4/E3*, and *E4/E4*. *P *values for a linear trend for *E2, E3* and *E4*.

**Table 4:** Odds ratios (ORs) and 95% confidence intervals (CIs) for past history of selected chronic diseases according APOE genotypes and gender.

Diseases	Genotype	Females			Males			*P* values for interaction
		Yes/No	OR	95% CI	Yes/No	OR	95% CI	
Myocardial infarction	*E2*	4/129	0.52	0.17–1.62	12/94	1.70	0.81–3.56	
	*E3*	22/475	1.00	—	29/335	1.00	—	
	*E4*	21/205	1.93	1.00–3.70	22/144	1.92	1.03–3.58	
	*E4* versus * E2E3 *		2.18	1.16–4.09		1.68	0.94–3.00	
		*P* = 0.009			*P* = 0.463			0.008

Stroke	*E2*	9/124	1.34	0.57–3.15	6/100	1.22	0.42–3.55	
	*E3*	21/476	1.00	—	16/348	1.00	—	
	*E4*	13/213	1.11	0.53–2.33	9/157	1.05	0.42–2.61	
	*E4* versus *E2E3 *		1.03	0.51–2.06		1.00	0.42–2.38	
		*P* = 0.772			*P* = 0.846			0.850

Diabetes	*E2*	22/111	0.99	0.56–1.73	14/92	0.93	0.47–1.87	
	*E3*	68/429	1.00	—	53/311	1.00	—	
	*E4*	32/194	0.89	0.55–1.44	15/151	0.50	0.26–0.97	
	*E4* versus *E2E3 *		0.89	0.56–1.42		0.51	0.27–0.97	
		*P* = 0.706			*P* = 0.103			0.762

Gallbladder disease	*E2*	31/102	1.28	0.79–2.08	5/101	0.58	0.22–1.58	
	*E3*	92/405	1.00	—	29/335	1.00	—	
	*E4*	31/195	0.67	0.43–1.06	15/151	1.14	0.58–2.26	
	*E4* versus *E2E3 *		0.64	0.41–0.99		1.27	0.66–2.46	
		*P* = 0.025			*P* = 0.244			0.088

ORs were adjusted for age, race, educational levels, cigarette smoking (pack-years), alcohol intake, body mass index, energy-adjusted dietary saturated fat, cholesterol, fiber and total calcium intakes, and family size. *E2* includes *E2/E2*, *E2/E3*, and *E2/E4*, and *E4* includes *E4/E3* and *E4/E4*. *P *values for a linear trend for *E2, E3* and *E4*.
